# Crystal structures of two bi­cyclo­[5.1.0]octa­nes: potassium *trans*-bi­cyclo­[5.1.0]octane-4-carboxyl­ate monohydrate and *cis*-bi­cyclo­[5.1.0]octan-4-yl 4-bromo­benzene­sulfonate

**DOI:** 10.1107/S2056989017011756

**Published:** 2017-08-21

**Authors:** Peter W. R. Corfield, Richard A. Kershaw

**Affiliations:** a Department of Chemistry, Fordham University, 441 East Fordham Road, Bronx, NY 10458, USA; b Department of Chemistry, The Ohio State University, Columbus, Ohio 43210, USA

**Keywords:** crystal structure, bicyclic, *trans*-fused, *cis*-fused, octa­ne

## Abstract

The crystal structures of the *trans*-fused compound potassium *trans*-bi­cyclo­[5.1.0]octane-4-carboxyl­ate monohydrate, K^+^·C_9_H_13_O_2_^−^·H_2_O, (I), and of *cis*-bi­cyclo­[5.1.0]octan-4-yl 4-bromo­benzene­sulfonate, C_14_H_17_BrO_3_S, (II), have been determined. Compound (I) represents the smallest *trans*-fused cyclo­propane structure known to date, and features the expected *shortening* of the bridging C—C bond relative to the other cyclo­propane bond lengths, in contrast to the *cis*-fused system, (II), where all of the cyclo­propane bond lengths are the same. The bicyclic ring system of (I) is disordered across a crystallographic mirror plane. The geometries of the *cis*-fused and *trans*-fused ring systems are compared.

## Abstract 2

The geometry of potassium *trans*-bi­cyclo­[5.1.0]octane-4-carboxyl­ate monohydrate, a highly strained *trans*-fused bicyclic ring system, is compared with that of a less-strained *cis*-bi­cyclo­[5.1.0]octane derivative.

## Chemical context   

Extensive studies on the reactivities of the bridge bond in *trans*-fused bicyclic cyclo­propane derivatives (Gassman *et al.*, 1968 ▸) led to proposal of the ‘twist’-bent bond to describe the bonding in these [5.1.0] bicyclic systems (Gassman, 1967 ▸). The [5.1.0]octa­nes are expected to be more highly strained than the corresponding *trans*-fused bi­cyclo­[4.2.0]octa­nes which had previously been prepared (Cava & Moroz, 1962 ▸). Our studies were initiated in order to illuminate discussions of bonding by providing accurate geometric parameters for the most strained systems available. Several 4-substituted derivatives of *trans*-fused bicyclic [5.1.0]octa­nes were studied, but in most, disordering of the mol­ecules in the crystal precluded any refined structure that would give useful information. Even the *trans*-fused bicyclic [5.1.0]octane 4-carboxyl­ate structure presented here is disordered, but we were able to determine a reasonable geometry for the bicyclic system. The structure of a 4-substituted *cis*-fused bicyclic [5.1.0]octane was also determined, so that a comparison of the ring geometries could be made. These studies formed part of the MS and PhD theses of one of us (Kershaw, 1972 ▸, 1974 ▸), and were presented at the 1973 winter meeting of The American Crystallographic Association.

## Structural commentary   

**Figure 1:**
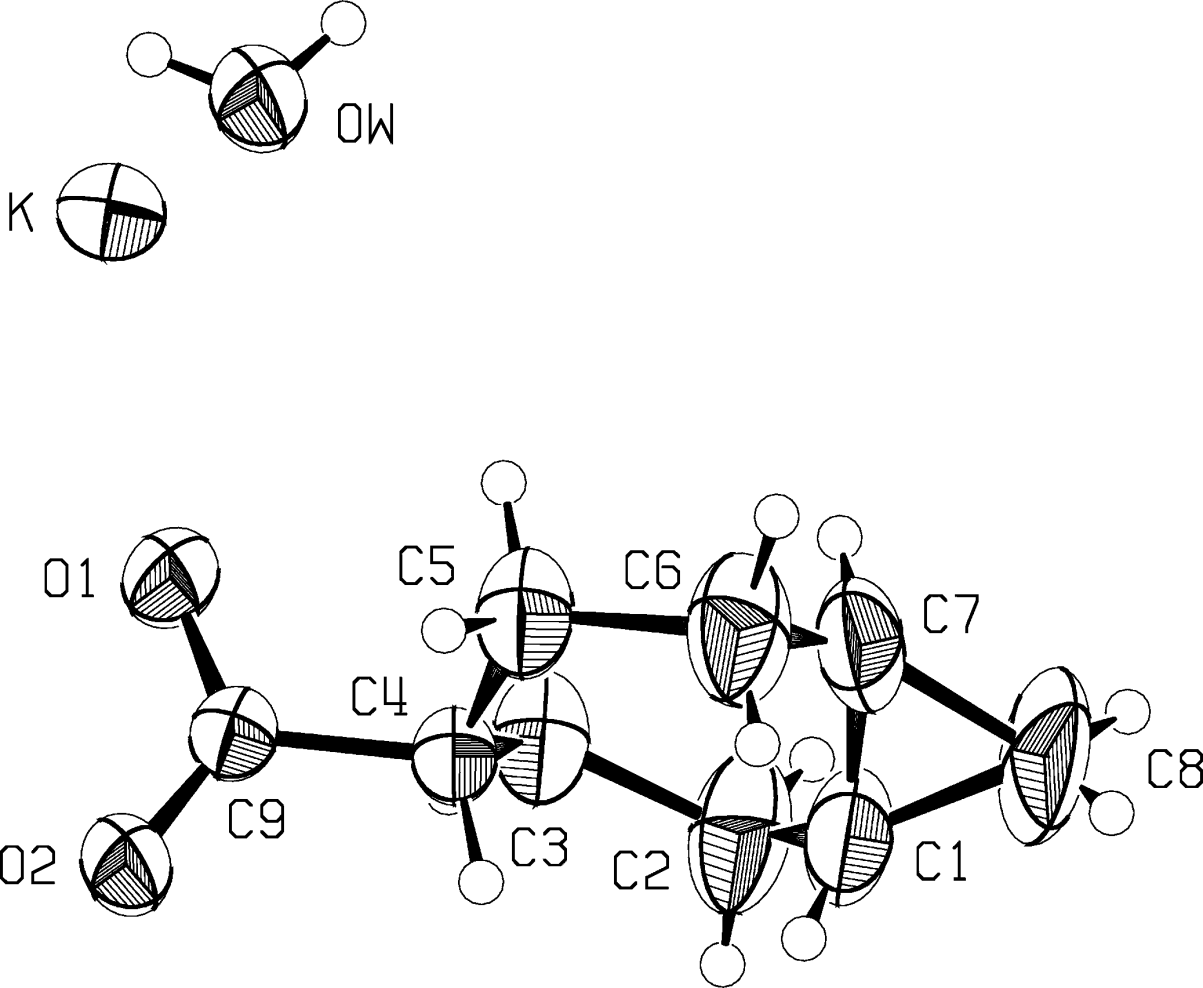
The asymmetric unit of compound (I). Displacement ellipsoids are at the 50% probability level. Sizes of the H atoms are arbitrary.

**Figure 3:**
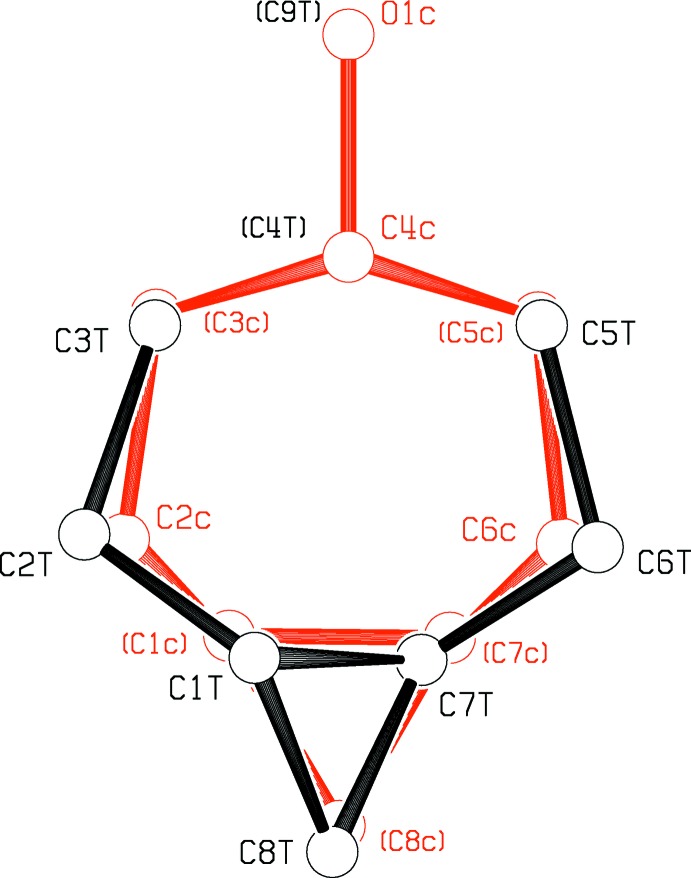
A superposition of the ring systems found for (I) and (II), viewed normal to the planes through C3, C4 and C5. The *trans*-fused structure is in black and the *cis*-fused structure in red.

Table 1 ▸ presents a comparison of the geometries of the *trans*-fused [5.1.0] (I) and *cis*-fused [5.1.0] (II) octane rings. Figs. 1 ▸ and 2 ▸ show the asymmetric units of the two mol­ecules, while Figs. 3 ▸ and 4 ▸ show the *cis*- and *trans*-fused rings superimposed upon one another. It can be seen that in the *cis*-fused system (II), chemically equivalent bonds and angles are the same, and so are the torsional angles. Thus the *cis*-fused compound has an excellent, non-crystallographic mol­ecular mirror plane.
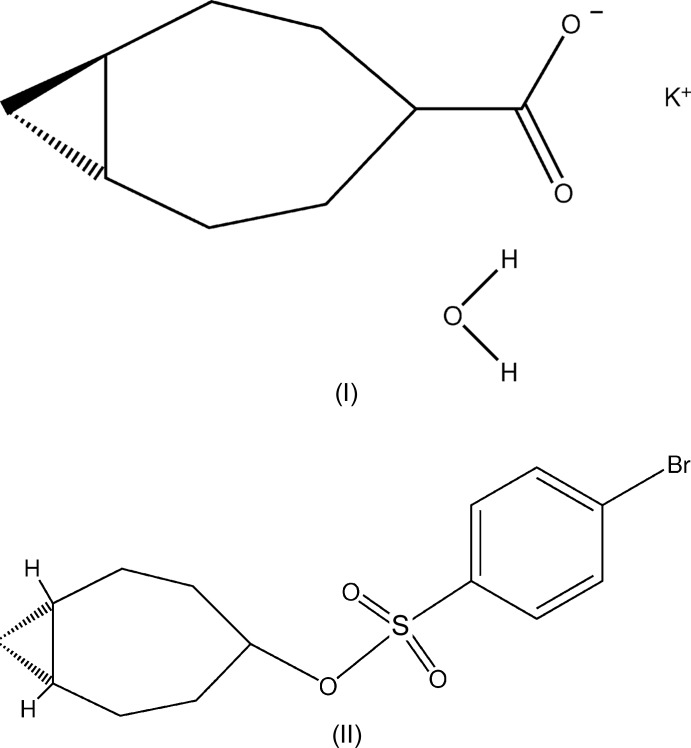


In contrast, while the *trans*-fused derivative cannot have a mol­ecular mirror plane; the mol­ecule sits astride a crystallographic mirror plane, probably due to the packing requirements of the potassium cation and the carboxyl­ate part of the mol­ecule, and necessarily leading to a disordered structure. Treatment of the disorder is discussed in the *Refinement* section. One of the assumptions made in the refinements of (I) was that chemically equivalent bonds and angles would be the same, so it was important to verify that this was the case in the *cis*-fused compound, (II). In both structures, the substituent on C4 is in the *exo* position. In (I), the plane of the carboxyl­ate substituent on C4 is necessarily at 90° to the mol­ecular plane through C2, C3, C5 and C6, while in (II) the roughly planar set C4, O1, S and C11 is tilted at 71.9 (2)° to the mol­ecular plane and at 49.6 (1)° to the plane through the phenyl group. In both structures, displacement ellipsoids for the cyclo­propane methyl­ene group indicate motion perpendicular to the cyclo­propane ring.

**Figure 4:**
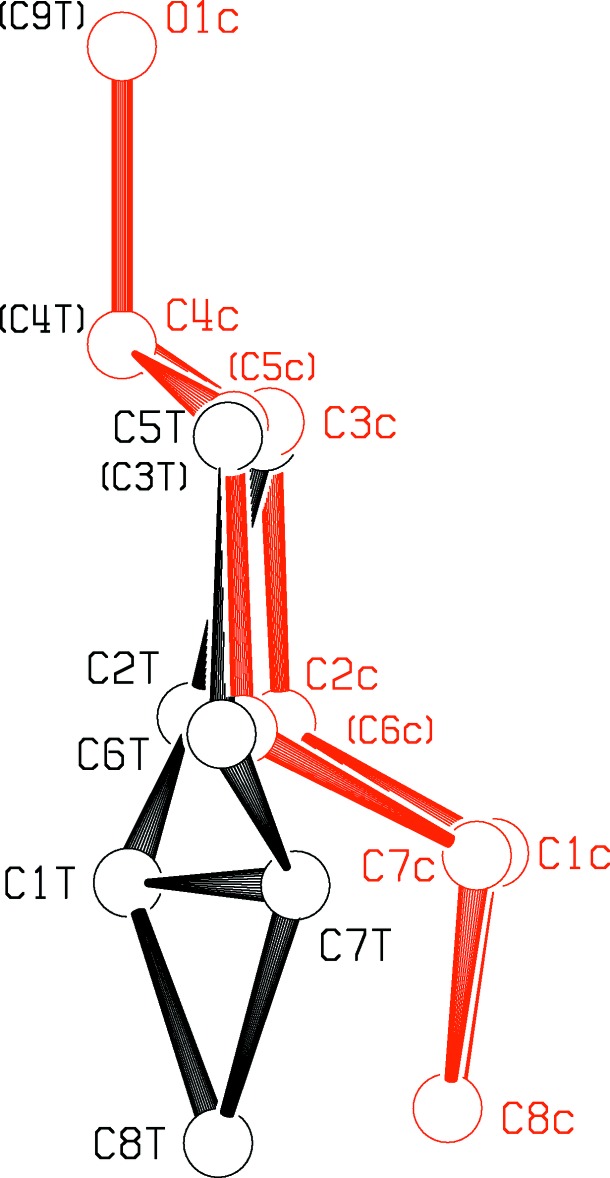
A view of the superposition of (I) and (II) at 90° to that in Fig. 3 ▸.

The two bicyclic systems are rather similar in the top view given in Fig. 3 ▸. *trans*-Fusion changes the conformation angles around C2—C3 and C5—C6, as seen in Fig. 4 ▸ and in Table 1 ▸. Fig. 3 ▸ shows that the *trans*-fusion is also accommodated by expansion of the angles at C3 and C5 from an average of 112.7 (2) to 117.5 (8)°, contraction of the angles at C2 and C6 from an average of 113.1 (3) to 107.1 (4)°, an increase in the external angles at C1 and C7 to 130.4 (8) from an average of 121.3 (3)°, and a lengthening of bonds C3—C4 and C5—C4 from 1.505 (4) to 1.538 (4) Å. The H1⋯H7 distance of 2.32 Å in (II) is increased to 2.84 Å in the *trans*-fused (I) structure. There is a significant shortening of the bridgehead bond C1—C7 in the *trans*-fused compound, from 1.493 (5) Å in (II) to 1.463 (6) Å in (I), which leads to a distortion of the cyclo­propane ring from equilateral triangular geometry, with reduction of the angle at C8 from 60.0 (2)° in (II) to 58.4 (3)° in (I). Such shortening of the strained twist-bent bond, though counter-intuitive, was expected (Kershaw, 1974 ▸, p2), because much of the electron density of the bond would lie outside the inter­nuclear line. We carried out geometry optimization of both *trans*- and *cis*-fused C_8_H_14_ systems using B3LYP density functional calculations (*GAUSSIAN09*; Frisch *et al.*, 2013 ▸), with results that also showed the trends noted above, including a calculated shortening of the bridgehead C1—C7 bond length by 0.014 Å.

## Supra­molecular features   

**Figure 5:**
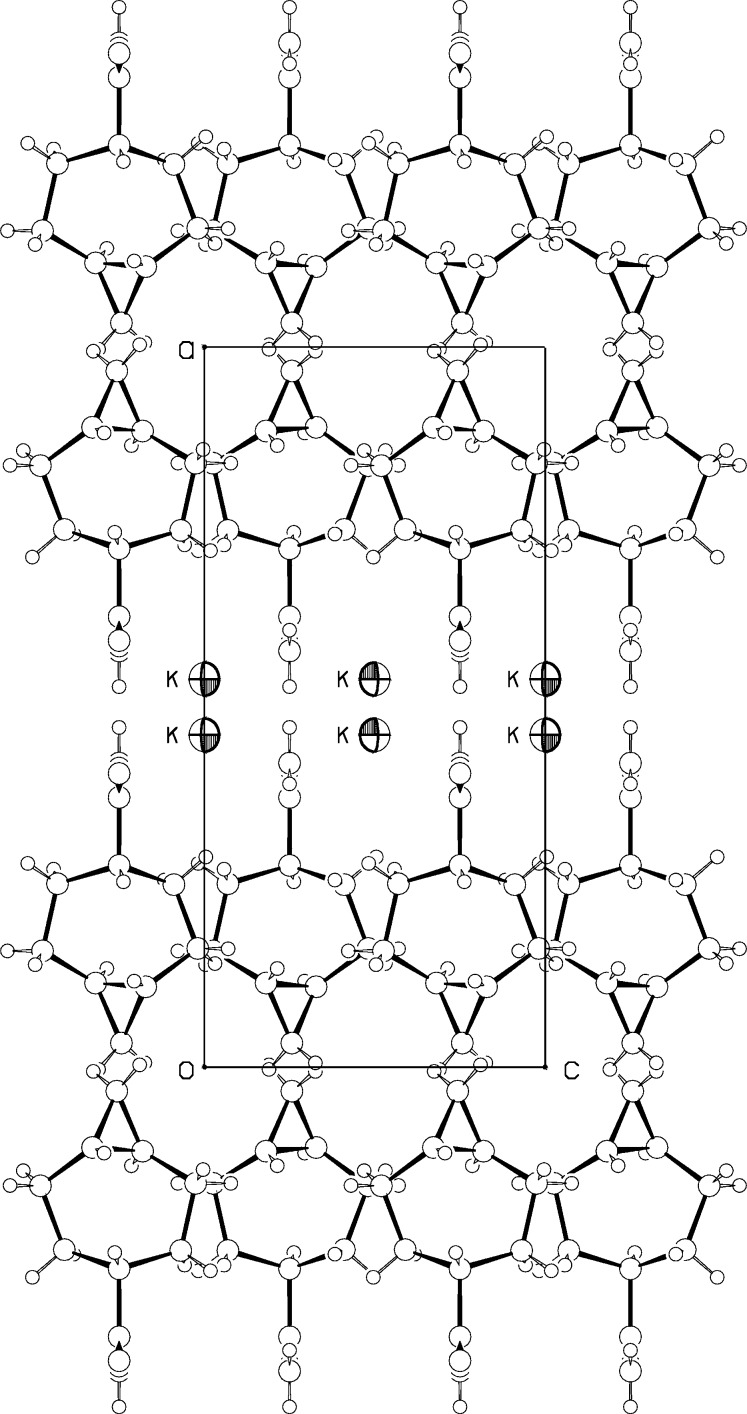
Projection of (I) down the *b* axis. Disordered [5.1.0]octane moieties related by the mirror at *z* = 0.25 are not shown.

**Figure 6:**
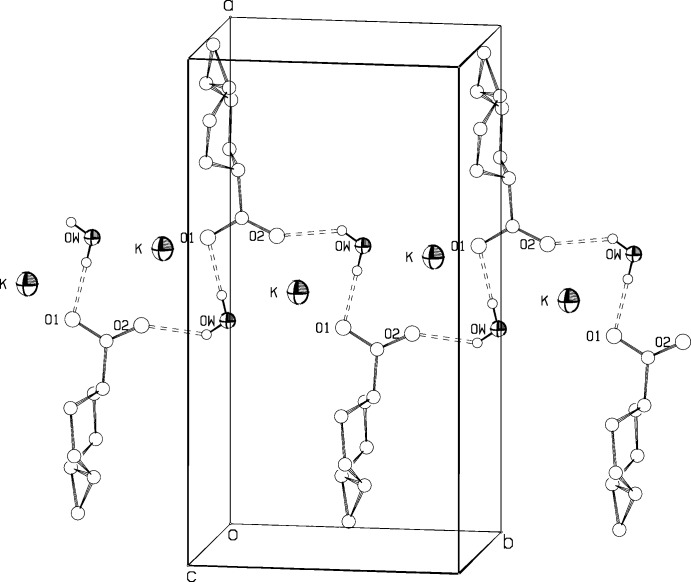
One of the two hydrogen-bonded chains parallel to the *b* axis in (I).

Fig. 5 ▸ gives a packing diagram for (I). There are alternating layers of hydro­phobic inter­actions between the cyclo­propane ends of the mol­ecules and of charge inter­actions between the carboxyl­ate ends of the mol­ecules and the potassium ions. In addition, the water mol­ecules in (I) form strong hydrogen bonds (Table 2 ▸) to carboxyl­ate oxygen atoms of two separate [5.1.0] octane mol­ecules, linking the anions into chains parallel to the *b* axis, as can be seen in Fig. 6 ▸. The hydrogen-bond lengths are rather short, with O3—H⋯O1(*x* − 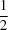, 1 − *y*, *z*) = 2.701 (3) Å and O3—H⋯O2(*x* − 1, *y*, *z*) = 2.757 (4) Å. The water O atoms may lie slightly off the mirror plane at *y* =1/4, as indicated by the displacement ellipsoid values, which would change the hydrogen-bond geometry a little. Strong hydrogen bonds are consistent with retention of the water of hydration even after recrystallization from a non-aqueous solvent, and also with the shifts in O—H stretching frequencies in the IR to 3060 and 3360 cm^−1^. The potassium ions lie in between two of the hydrogen-bonded chains, and have four carboxyl­ate and two water oxygen atoms as near neighbors, in a distorted flattened trigonal–prismatic array, with K—O distances ranging from 2.719 (3) to 2.879 (3) Å.

**Figure 7:**
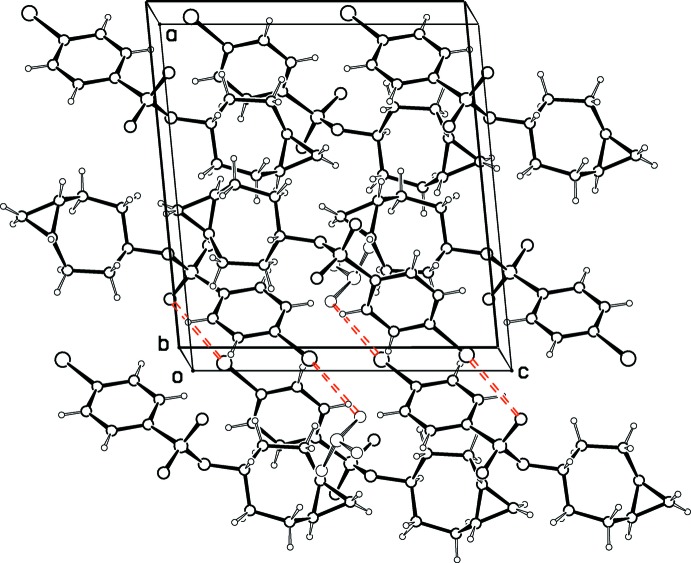
Packing diagram for (II), showing halogen bonds in red.

The supra­molecular structure for (II) features the presence of inter­molecular halogen bonds between Br and O2 (Fig. 7 ▸), which link mol­ecules related by the screw axes at *x* = 0 into a helical arrangement. The Br ⋯ O2(2 − *x*, *y* − 1/2, −*z* − 1/2) distance is 3.230 (2) Å, which is 96% of the sum of the van der Waals radii, while the C14—Br ⋯ O2 and Br ⋯ O2—C9 angles are 170.06 (8) and 107.81 (9)°, respectively. These parameters are consistent with moderate halogen bonding according to a systematic study of such inter­molecular inter­actions in the CSD (Lommerse *et al.*, 1996 ▸). Also, a review of the role of halogen bonding in crystal engineering (Metrangolo *et al.*, 2005 ▸), stresses the importance in halogen bonding of the aromatically bound bromine seen in the present compound. There are no other inter­molecular contacts of note and the shortest H⋯H contact is H3*A* ⋯ H8*B*(*x*, 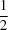 − *y*, *z* − 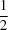), at 2.47 Å.

## Database survey   

Of 399 hits in the Cambridge Structure Database (CSD, Version 5.35; Groom *et al.*, 2016 ▸) for the [5.1.0] ring system, 105 have 3D coordinates available, unsubstituted H atoms at the bridgehead positions, and conventional *R* factors of 0.05 or less, leading to 244 [5.1.0] geometries. All of the systems are *cis*-fused; no *trans*-fused [5.1.0] system was found. The average geometry of the CSD bicyclic ring systems displays the same near-perfect mirror symmetry found in the present *cis*-fused structure. The geometrical parameters of the *cis*-fused system described here do not differ significantly from the database geometries. In particular, the average bridgehead C—C bond length in the CSD set does not differ significantly from the other cyclo­propane bond lengths, just as in the present *cis*-fused structure, (II), and in contrast to the *trans*-fused structure, (I), where the bridgehead C—C bond length is shortened. Both the current *cis*-structure and the ensemble of [5.1.0] structures show the significant lengthening of bonds C2—C3 and C5—C6 relative to other bonds in the ring system noted in Table 1 ▸.

Searches for simple bicyclic [6.1.0] systems yielded only 14 hits. Two of these were *trans*-fused structures, (Szabo *et al.*, 1973 ▸; Hayes *et al.*, 2005 ▸), with H1⋯H7 distances of 2.80 and 2.95 Å, respectively. In both structures, the bridgehead C—C bond length was longer by 0.03 Å than the other two cyclo­propane C—C bond lengths, in contrast to the shorter bridgehead C—C bond observed in (I).

## Synthesis and crystallization   

Syntheses of these ring systems are described in Gassman *et al.* (1971 ▸). Samples of *trans*-fused bi­cyclo [5.1.0] octane 4-carb­oxy­lic acid and crystals of the *cis*-bi­cyclo­[5.1.0]octan-4-yl 4-bromo­benzene­sulfonate were supplied by Dr Paul G. Gassman. The *trans*-fused acid was titrated with potassium hydroxide, and crystals of the potassium salt were obtained by evaporation to dryness and recrystallization from a benzene–methanol mixture. Analysis: C 50.89%, H 7.15%, in good agreement with calculated values of C 51.40% and H 7.19% for K[C_9_O_2_H_13_]·H_2_O.

## Refinement   

Crystal data, data collection and structure refinement details are summarized in Table 3 ▸. For the *trans*-fused structure (I), only one octant of data was collected. Also in (I), reflections with *I*<2σ were not saved when the data were processed. These weak reflections were later patched back into the structure factor file, with intensities set at σ(*I*), where σ(*I*) was the average value for reflections at a similar θ value for weak reflections in the data set with 2σ<*I*<3σ. It became apparent, however, that most of the missing reflections were higher order. We chose to use a cut-off value of 0.82 for the resolution of reflections used in final refinements, as about 50% of the intensities at this resolution were above 3σ, while only 11% of the reflections at resolutions above this value had *I*>2σ.

After extensive efforts, it was concluded that the near-perfect mirror symmetry in (I) apart from C1 and C7 hampered successful refinement in the non-centrosymmetric space group *Pca*2_1_. Accordingly, all further refinements were carried out assuming a disordered structure in space group *Pbcm*. Initially, only atoms C1 and C7 were disordered, but it became apparent that bonded atoms C2 and C6 should be refined individually, and that C8 should also be allowed to move off the mirror plane at *z* = 0.25. Later, atoms C3 and C5 were also refined individually. It was necessary to impose tight restraints on the geometry to overcome the high correlation between parameters for C2 and C3 and the reflected images of C5 and C6. This was done by tightly restricting differences between chemically equivalent bond lengths and angles on either side of the octane ring.

No special measures were necessary in the refinement of (II).

In both compounds, C-bound H atoms were constrained to idealized positions, with C—H distances of 0.97 Å for CH_2_ groups, 0.98 Å for methine CH groups and 0.93 Å for aromatic H atoms, and with *U*_eq_ values set at 1.3 times the *U*_iso_ of their bonded atoms for the CH_2_ H atoms, and 1.2 times for methine and aromatic H atoms. In (I), H1 and H7 were initially refined independently, in case their positions could throw light on the twist-bent bond, but as they refined into positions indistinguishable from the constrained positions, they were constrained in the final refinements. The water H atoms in (I) were found in a difference-Fourier map, and their positional coordinates were refined whilst their *U*_eq_ values set at 1.3 times the *U*_iso_ of the O atom. As a check, the *U*_eq_ values for these H atoms were allowed to vary, but as there was no appreciable change in these *U* values, they were constrained in the final refinement.

## Supplementary Material

Crystal structure: contains datablock(s) I, II. DOI: 10.1107/S2056989017011756/pk2603sup1.cif

Structure factors: contains datablock(s) II. DOI: 10.1107/S2056989017011756/pk2603IIsup3.hkl

CCDC references: 1568784, 1568785

Additional supporting information:  crystallographic information; 3D view; checkCIF report

## Figures and Tables

**Figure 2:**
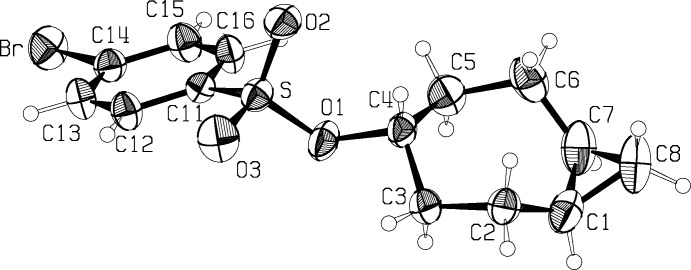
The asymmetric unit of compound (II). Displacement ellipsoids are at the 50% probability level. Sizes of the H atoms are arbitrary.

**Table 1:** Selected bond lengths, angles, and conformational angles (Å, °), for (I) and (II)

	(I) (*trans*)	(II) (*cis*)		(I) (*trans*)	(II) (*cis*)
C1—C2	1.513 (4)	1.485 (4)	C7—C1—C2	112.9 (5)	119.5 (3)
C6—C7	1.514 (4)	1.502 (5)	C6—C7—C1	112.8 (5)	119.6 (3)
					
C2—C3	1.543 (4)	1.534 (3)	C1—C2—C3	107.1 (3)	112.9 (2)
C5—C6	1.543 (3)	1.542 (4)	C5—C6—C7	107.1 (3)	113.3 (3)
					
C3—C4	1.538 (3)	1.510 (4)	C2—C3—C4	117.8 (6)	113.1 (2)
C5—C4	1.538 (4)	1.500 (4)	C6—C5—C4	117.2 (4)	112.2 (2)
			C3—C4—C5	118.0 (3)	118.5 (2)
					
			C2—C1—C8	130.5 (4)	121.6 (3)
			C6—C7—C8	130.4 (4)	120.9 (3)
C1—C8	1.500 (4)	1.499 (5)	C7—C1—C8	60.82 (14)	59.7 (2)
C7—C8	1.500 (4)	1.489 (5)	C1—C7—C8	60.80 (14)	60.3 (2)
C1—C7	1.463 (6)	1.493 (5)	C1—C8—C7	58.4 (3)	60.0 (2)
					
			C1—C2—C3—C4	−28.1 (12)	−81.7 (3)
			C7—C6—C5—C4	46.4 (12)	80.7 (4)
					
			C2—C3—C4—C5	82.2 (8)	64.4 (3)
			C6—C5—C4—C3	−66.4 (8)	−63.4 (4)
					
			C3—C2—C1—C7	−53.6 (9)	66.1 (4)
			C5—C6—C7—C1	−75.1 (8)	−67.2 (4)
					
			C2—C1—C7—C6	110.5 (5)	0.6 (5)
			C2—C1—C7—C8	−124.8 (3)	111.6 (4)
			C6—C7—C1—C8	−124.8 (3)	−110.9 (4)

**Table 2:** Hydrogen-bond geometry (Å, °) for (I)

*D*—H⋯*A*	*D*—H	H⋯*A*	*D*⋯*A*	*D*—H⋯*A*
O*W*—H*WA*⋯O1^i^	0.82 (1)	1.88 (1)	2.701 (3)	180 (4)
O*W*—H*WB*⋯O2^ii^	0.82 (1)	2.08 (3)	2.757 (4)	140 (3)

Symmetry codes: (i) 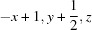; (ii) 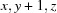.

**Table 3:** Experimental details

	(I)	(II)
Crystal data		
Chemical formula	K^+^·C_9_H_13_O_2_^−^·H_2_O	C_14_H_17_BrO_3_S
*M*_r_	210.31	345.24
Crystal system, space group	Orthorhombic, *P* *b* *c* *m*	Monoclinic, *P*2_1_/*c*
Temperature (K)	297	297
*a*, *b*, *c* (Å)	16.148 (13), 8.631 (9), 7.674 (10)	12.829 (1), 9.759 (1), 11.730 (2)
α, β, γ (°)	90, 90, 90	90, 95.74 (1), 90
*V* (Å^3^)	1070 (2)	1461.2 (3)
*Z*	4	4
Radiation type	Mo *K*α	Cu *K*α
μ (mm^−1^)	0.47	5.19
Crystal size (mm)	0.5 × 0.4 × 0.1	0.29 × 0.24 × 0.18

Data collection		
Diffractometer	Picker four-circle	Picker four-circle
Absorption correction	Gaussian (Busing & Levy, 1957 ▸)	Gaussian (Busing & Levy, 1957 ▸)
*T*_min_, *T*_max_	0.842, 0.954	0.267, 0.456
No. of measured, independent and observed [*I* > 2σ(*I*)] reflections	1926, 1104, 795	2447, 2381, 2154
*R*_int_	0.02	0.02
(sin θ/λ)_max_ (Å^−1^)	0.735	0.580

Refinement		
*R*[*F* ^2^ > 2σ(*F* ^2^)], *wR*(*F* ^2^), *S*	0.034, 0.096, 1.00	0.027, 0.091, 1.08
No. of reflections	1104	2381
No. of parameters	98	173
No. of restraints	16	0
H-atom treatment	H atoms treated by a mixture of independent and constrained refinement	H-atom parameters constrained
Δρ_max_, Δρ_min_ (e Å^−3^)	0.15, −0.17	0.34, −0.31

Data reduction followed procedures in Corfield *et al.* (1973 ▸), with *p* = 0.05 for (I) and 0.06 for (II). Computer programs: *SHELXL2014* (Sheldrick, 2015 ▸), *ORTEPIII* (Burnett & Johnson, 1996 ▸) and local superposition program (Corfield, 1972 ▸).

## supplementary crystallographic information

### Potassium trans-bicyclo[5.1.0]octane-4-carboxylate monohydrate (I) Crystal data

**Table:**

K^+^·C_9_H_13_O_2_^−^·H_2_O	*F*(000) = 448
*M**_r_* = 210.31	*D*_x_ = 1.306 Mg m^−^^3^*D*_m_ = 1.345 (11) Mg m^−^^3^*D*_m_ measured by Flotation in benzene–carbon tetrachloride mixtures
Orthorhombic, *P**b**c**m*	Mo *K*α radiation, λ = 0.7107 Å
*a* = 16.148 (13) Å	Cell parameters from 19 reflections
*b* = 8.631 (9) Å	µ = 0.47 mm^−^^1^
*c* = 7.674 (10) Å	*T* = 297 K
*V* = 1070 (2) Å^3^	Plate, colorless
*Z* = 4	0.5 × 0.4 × 0.1 mm

### Potassium trans-bicyclo[5.1.0]octane-4-carboxylate monohydrate (I) Data collection

**Table:**

Picker four-circle diffractometer	795 reflections with *I* > 2σ(*I*)
Radiation source: sealed X-ray tube	*R*_int_ = 0.02
Oriented graphite 200 reflection monochromator	θ_max_ = 31.5°, θ_min_ = 1.3°
θ/2θ scans	*h* = 0→23
Absorption correction: gaussian Busing & Levy (1957)	*k* = 0→12
*T*_min_ = 0.842, *T*_max_ = 0.954	*l* = 0→11
1926 measured reflections	9 standard reflections every 220 reflections
1104 independent reflections	intensity decay: −3.0(8)

### Potassium trans-bicyclo[5.1.0]octane-4-carboxylate monohydrate (I) Refinement

**Table:**

Refinement on *F*^2^	Primary atom site location: heavy-atom method
Least-squares matrix: full	Secondary atom site location: real-space vector search
*R*[*F*^2^ > 2σ(*F*^2^)] = 0.034	Hydrogen site location: mixed
*wR*(*F*^2^) = 0.096	H atoms treated by a mixture of independent and constrained refinement
*S* = 1.00	*w* = 1/[σ^2^(*F*_o_^2^) + 0.250*P*] where *P* = (*F*_o_^2^ + 2*F*_c_^2^)/3
1104 reflections	(Δ/σ)_max_ = 0.002
98 parameters	Δρ_max_ = 0.15 e Å^−^^3^
16 restraints	Δρ_min_ = −0.17 e Å^−^^3^

### Potassium trans-bicyclo[5.1.0]octane-4-carboxylate monohydrate (I) Special details

**Table:**

Geometry. All esds (except the esd in the dihedral angle between two l.s. planes) are estimated using the full covariance matrix. The cell esds are taken into account individually in the estimation of esds in distances, angles and torsion angles; correlations between esds in cell parameters are only used when they are defined by crystal symmetry. An approximate (isotropic) treatment of cell esds is used for estimating esds involving l.s. planes.

### Potassium trans-bicyclo[5.1.0]octane-4-carboxylate monohydrate (I) Fractional atomic coordinates and isotropic or equivalent isotropic displacement parameters (Å^2^)

**Table:**

	*x*	*y*	*z*	*U*_iso_*/*U*_eq_	Occ. (<1)
K1	0.46108 (4)	0.2500	0.5000	0.0513 (2)	
OW	0.42215 (13)	0.4712 (2)	0.7500	0.0625 (6)	
HWA	0.4717 (8)	0.493 (4)	0.7500	0.081*	
HWB	0.3937 (19)	0.550 (3)	0.7500	0.081*	
O1	0.41518 (12)	0.0440 (2)	0.7500	0.0581 (6)	
O2	0.40765 (12)	−0.2105 (2)	0.7500	0.0603 (6)	
C1	0.1115 (2)	−0.0466 (5)	0.8279 (6)	0.0760 (17)	0.5
H1	0.1194	−0.1551	0.7941	0.091*	0.5
C2	0.1648 (4)	0.0018 (15)	0.9808 (6)	0.0853 (9)	0.5
H2A	0.1645	−0.0781	1.0697	0.111*	0.5
H2B	0.1439	0.0970	1.0315	0.111*	0.5
C3	0.2534 (3)	0.0263 (16)	0.9108 (9)	0.0640 (19)	0.5
H3A	0.2597	0.1353	0.8830	0.083*	0.5
H3B	0.2918	0.0032	1.0044	0.083*	0.5
C4	0.28013 (16)	−0.0677 (3)	0.7500	0.0449 (7)	
H4	0.2575	−0.1725	0.7626	0.054*	0.5
C5	0.2547 (3)	−0.0071 (16)	0.5693 (8)	0.0640 (19)	0.5
H5A	0.2829	−0.0691	0.4821	0.083*	0.5
H5B	0.2749	0.0982	0.5579	0.083*	0.5
C6	0.1614 (4)	−0.0070 (14)	0.5256 (6)	0.0853 (9)	0.5
H6A	0.1509	0.0549	0.4224	0.111*	0.5
H6B	0.1423	−0.1118	0.5038	0.111*	0.5
C7	0.1167 (2)	0.0611 (5)	0.6811 (6)	0.0819 (19)	0.5
H7	0.1369	0.1641	0.7140	0.098*	0.5
C8	0.0335 (2)	0.0232 (6)	0.7568 (9)	0.115 (2)	0.5
H8A	0.0060	0.1021	0.8258	0.149*	0.5
H8B	−0.0031	−0.0430	0.6897	0.149*	0.5
C9	0.37448 (16)	−0.0812 (3)	0.7500	0.0418 (6)	

### Potassium trans-bicyclo[5.1.0]octane-4-carboxylate monohydrate (I) Atomic displacement parameters (Å^2^)

**Table:**

	*U*^11^	*U*^22^	*U*^33^	*U*^12^	*U*^13^	*U*^23^
K1	0.0584 (4)	0.0468 (3)	0.0486 (4)	0.000	0.000	0.0004 (3)
OW	0.0479 (13)	0.0488 (12)	0.0906 (17)	0.0055 (10)	0.000	0.000
O1	0.0438 (11)	0.0462 (11)	0.0842 (16)	−0.0067 (9)	0.000	0.000
O2	0.0428 (10)	0.0413 (11)	0.0967 (18)	0.0057 (8)	0.000	0.000
C4	0.0361 (13)	0.0468 (15)	0.0517 (17)	0.0021 (12)	0.000	0.000
C9	0.0377 (13)	0.0462 (15)	0.0416 (15)	−0.0013 (13)	0.000	0.000
C1	0.037 (2)	0.109 (4)	0.081 (4)	0.003 (3)	0.003 (2)	0.010 (4)
C2	0.0528 (13)	0.139 (3)	0.0644 (17)	0.0065 (17)	0.0150 (13)	−0.0114 (17)
C3	0.0470 (11)	0.096 (5)	0.0494 (17)	0.0065 (15)	−0.0001 (11)	−0.003 (3)
C5	0.0470 (11)	0.096 (5)	0.0494 (17)	0.0065 (15)	0.0001 (11)	0.003 (3)
C6	0.0528 (13)	0.139 (3)	0.0644 (17)	0.0065 (17)	−0.0150 (13)	0.0114 (17)
C7	0.043 (2)	0.111 (4)	0.092 (5)	0.018 (3)	−0.006 (2)	0.026 (4)
C8	0.046 (2)	0.169 (5)	0.128 (5)	0.020 (3)	0.042 (6)	0.002 (14)

### Potassium trans-bicyclo[5.1.0]octane-4-carboxylate monohydrate (I) Geometric parameters (Å, º)

**Table:**

K1—O1	2.719 (3)	C2—H2A	0.9700
K1—O1^i^	2.719 (3)	C2—H2B	0.9700
K1—OW	2.779 (3)	C3—C4	1.538 (3)
K1—OW^i^	2.779 (3)	C3—H3A	0.9700
K1—O2^ii^	2.879 (3)	C3—H3B	0.9700
K1—O2^iii^	2.879 (3)	C4—C9	1.528 (4)
K1—K1^iv^	3.837 (5)	C4—C5	1.538 (4)
K1—HWA	2.85 (2)	C4—H4	0.9800
OW—K1^v^	2.779 (3)	C5—C6	1.543 (3)
OW—HWA	0.822 (10)	C5—H5A	0.9700
OW—HWB	0.818 (10)	C5—H5B	0.9700
O1—C9	1.264 (3)	C6—C7	1.513 (4)
O2—C9	1.238 (3)	C6—H6A	0.9700
C1—C7	1.463 (6)	C6—H6B	0.9700
C1—C8	1.500 (4)	C7—C8	1.500 (4)
C1—C2	1.513 (4)	C7—H7	0.9800
C1—H1	0.9800	C8—H8A	0.9700
C2—C3	1.543 (3)	C8—H8B	0.9700
			
O1—K1—O1^i^	148.36 (9)	C4—C3—H3A	107.8
O1—K1—OW	84.29 (10)	C2—C3—H3A	107.8
O1^i^—K1—OW	88.63 (10)	C4—C3—H3B	107.8
O1—K1—OW^i^	88.63 (10)	C2—C3—H3B	107.8
O1^i^—K1—OW^i^	84.29 (10)	H3A—C3—H3B	107.2
OW—K1—OW^i^	153.85 (9)	C9—C4—C5	107.00 (18)
O1—K1—O2^ii^	78.92 (8)	C9—C4—C3	108.6 (2)
O1^i^—K1—O2^ii^	126.41 (7)	C5—C4—C3	118.0 (3)
OW—K1—O2^ii^	67.99 (7)	C9—C4—H4	107.6
OW^i^—K1—O2^ii^	135.07 (7)	C5—C4—H4	107.6
O1—K1—O2^iii^	126.41 (7)	C3—C4—H4	107.6
O1^i^—K1—O2^iii^	78.92 (8)	C4—C5—C6	117.2 (4)
OW—K1—O2^iii^	135.07 (7)	C4—C5—H5A	108.0
OW^i^—K1—O2^iii^	67.99 (7)	C6—C5—H5A	108.0
O2^ii^—K1—O2^iii^	85.18 (10)	C4—C5—H5B	108.0
K1—OW—K1^v^	87.32 (11)	C6—C5—H5B	108.0
K1—OW—HWA	86.3 (18)	H5A—C5—H5B	107.2
K1^v^—OW—HWA	86.3 (18)	C7—C6—C5	107.1 (3)
K1—OW—HWB	134.1 (7)	C7—C6—H6A	110.3
K1^v^—OW—HWB	134.1 (7)	C5—C6—H6A	110.3
HWA—OW—HWB	111 (4)	C7—C6—H6B	110.3
C9—O1—K1	134.46 (6)	C5—C6—H6B	110.3
K1—O1—K1^v^	89.76 (11)	H6A—C6—H6B	108.5
C9—O2—K1^vi^	115.16 (11)	C1—C7—C8	60.80 (14)
K1^vi^—O2—K1^iii^	83.57 (10)	C1—C7—C6	112.8 (5)
C7—C1—C8	60.82 (14)	C8—C7—C6	130.4 (4)
C7—C1—C2	112.9 (10)	C1—C7—H7	113.4
C8—C1—C2	130.4 (4)	C8—C7—H7	113.4
C7—C1—H1	113.3	C6—C7—H7	113.4
C8—C1—H1	113.3	C1—C8—C7	58.4 (3)
C2—C1—H1	113.3	C1—C8—H8A	117.9
C1—C2—C3	107.1 (3)	C7—C8—H8A	117.9
C1—C2—H2A	110.3	C1—C8—H8B	117.9
C3—C2—H2A	110.3	C7—C8—H8B	117.9
C1—C2—H2B	110.3	H8A—C8—H8B	115.1
C3—C2—H2B	110.3	O2—C9—O1	123.1 (2)
H2A—C2—H2B	108.5	O2—C9—C4	120.0 (2)
C4—C3—C2	117.8 (6)	O1—C9—C4	117.0 (2)
			
C1—C2—C3—C4	−28.1 (12)	C3—C2—C1—C7	−53.6 (9)
C7—C6—C5—C4	46.4 (12)	C2—C1—C7—C6	110.5 (5)
C2—C3—C4—C5	82.2 (8)	C2—C1—C7—C8	−124.8 (3)
C6—C5—C4—C3	−66.4 (8)	C6—C7—C1—C8	−124.8 (3)
C5—C6—C7—C1	−75.1 (8)		

Symmetry codes: (i) *x*, −*y*+1/2, −*z*+1; (ii) −*x*+1, *y*+1/2, *z*; (iii) −*x*+1, −*y*, −*z*+1; (iv) *x*, *y*, −*z*+1/2; (v) *x*, *y*, −*z*+3/2; (vi) −*x*+1, −*y*, *z*+1/2.

### Potassium trans-bicyclo[5.1.0]octane-4-carboxylate monohydrate (I) Hydrogen-bond geometry (Å, º)

**Table:**

*D*—H···*A*	*D*—H	H···*A*	*D*···*A*	*D*—H···*A*
O*W*—H*WA*···O1^ii^	0.82 (1)	1.88 (1)	2.701 (3)	180 (4)
O*W*—H*WB*···O2^vii^	0.82 (1)	2.08 (3)	2.757 (4)	140 (3)

Symmetry codes: (ii) −*x*+1, *y*+1/2, *z*; (vii) *x*, *y*+1, *z*.

### cis-Bicyclo[5.1.0]octan-4-yl 4-bromobenzenesulfonate (II) Crystal data

**Table:**

C_14_H_17_BrO_3_S	*F*(000) = 704
*M**_r_* = 345.24	*D*_x_ = 1.569 Mg m^−^^3^*D*_m_ = 1.566 Mg m^−^^3^*D*_m_ measured by density gradient column made from potassium tartrate and iodide solutions
Monoclinic, *P*2_1_/*c*	Cu *K*α radiation, λ = 1.5418 Å
*a* = 12.829 (1) Å	Cell parameters from 16 reflections
*b* = 9.759 (1) Å	θ = 30.0°
*c* = 11.730 (2) Å	µ = 5.19 mm^−^^1^
β = 95.74 (1)°	*T* = 297 K
*V* = 1461.2 (3) Å^3^	Block, colourless
*Z* = 4	0.29 × 0.24 × 0.18 mm

### cis-Bicyclo[5.1.0]octan-4-yl 4-bromobenzenesulfonate (II) Data collection

**Table:**

Picker four-circle diffractometer	2154 reflections with *I* > 2σ(*I*)
Radiation source: sealed X-ray tube	*R*_int_ = 0.02
Oriented graphite 200 reflection monochromator	θ_max_ = 63.4°, θ_min_ = 3.5°
θ/2θ scans	*h* = 0→14
Absorption correction: gaussian Busing & Levy (1957)	*k* = 0→11
*T*_min_ = 0.267, *T*_max_ = 0.456	*l* = −13→13
2447 measured reflections	3 standard reflections every 100 reflections
2381 independent reflections	intensity decay: 2.4(8)

### cis-Bicyclo[5.1.0]octan-4-yl 4-bromobenzenesulfonate (II) Refinement

**Table:**

Refinement on *F*^2^	Secondary atom site location: real-space vector search
Least-squares matrix: full	Hydrogen site location: inferred from neighbouring sites
*R*[*F*^2^ > 2σ(*F*^2^)] = 0.027	H-atom parameters constrained
*wR*(*F*^2^) = 0.091	*w* = 1/[σ^2^(*F*_o_^2^) + 0.430*P*] where *P* = (*F*_o_^2^ + 2*F*_c_^2^)/3
*S* = 1.08	(Δ/σ)_max_ = 0.003
2381 reflections	Δρ_max_ = 0.34 e Å^−^^3^
173 parameters	Δρ_min_ = −0.31 e Å^−^^3^
0 restraints	Extinction correction: SHELXL2014 (Sheldrick, 2015), Fc^*^=kFc[1+0.001xFc^2^λ^3^/sin(2θ)]^-1/4^
Primary atom site location: heavy-atom method	Extinction coefficient: 0.0062 (4)

### cis-Bicyclo[5.1.0]octan-4-yl 4-bromobenzenesulfonate (II) Special details

**Table:**

Geometry. All esds (except the esd in the dihedral angle between two l.s. planes) are estimated using the full covariance matrix. The cell esds are taken into account individually in the estimation of esds in distances, angles and torsion angles; correlations between esds in cell parameters are only used when they are defined by crystal symmetry. An approximate (isotropic) treatment of cell esds is used for estimating esds involving l.s. planes.

### cis-Bicyclo[5.1.0]octan-4-yl 4-bromobenzenesulfonate (II) Fractional atomic coordinates and isotropic or equivalent isotropic displacement parameters (Å^2^)

**Table:**

	*x*	*y*	*z*	*U*_iso_*/*U*_eq_	
Br	1.00989 (3)	0.32885 (3)	−0.39343 (3)	0.06640 (18)	
S	0.74088 (5)	0.62673 (6)	−0.03759 (5)	0.04479 (19)	
O1	0.69615 (16)	0.50385 (19)	0.02540 (14)	0.0595 (5)	
O2	0.81339 (16)	0.70034 (19)	0.03968 (16)	0.0591 (5)	
O3	0.65795 (18)	0.7021 (2)	−0.09750 (19)	0.0727 (6)	
C1	0.5504 (3)	0.3765 (3)	0.3453 (3)	0.0671 (8)	
H1	0.5006	0.3017	0.3269	0.081*	
C2	0.5300 (2)	0.5028 (3)	0.2761 (2)	0.0524 (6)	
H2A	0.5671	0.5788	0.3149	0.068*	
H2B	0.4557	0.5234	0.2708	0.068*	
C3	0.5639 (2)	0.4901 (3)	0.1548 (2)	0.0503 (6)	
H3A	0.5464	0.3991	0.1256	0.065*	
H3B	0.5248	0.5557	0.1052	0.065*	
C4	0.6796 (2)	0.5145 (3)	0.15002 (19)	0.0463 (6)	
H4	0.6967	0.6078	0.1767	0.056*	
C5	0.7557 (2)	0.4164 (4)	0.2114 (2)	0.0646 (8)	
H5A	0.8262	0.4406	0.1958	0.084*	
H5B	0.7412	0.3246	0.1822	0.084*	
C6	0.7495 (3)	0.4170 (4)	0.3420 (3)	0.0730 (9)	
H6A	0.8149	0.3823	0.3797	0.095*	
H6B	0.7413	0.5107	0.3671	0.095*	
C7	0.6607 (3)	0.3324 (3)	0.3782 (3)	0.0782 (10)	
H7	0.6722	0.2331	0.3777	0.094*	
C8	0.5957 (4)	0.3823 (4)	0.4680 (3)	0.0919 (12)	
H8A	0.6119	0.4718	0.5012	0.120*	
H8B	0.5715	0.3154	0.5205	0.120*	
C11	0.80882 (18)	0.5379 (2)	−0.13726 (19)	0.0399 (5)	
C12	0.7890 (2)	0.5658 (3)	−0.2526 (2)	0.0506 (6)	
H12	0.7356	0.6259	−0.2787	0.061*	
C13	0.8491 (2)	0.5039 (3)	−0.3287 (2)	0.0544 (7)	
H13	0.8372	0.5233	−0.4066	0.065*	
C14	0.92621 (19)	0.4138 (3)	−0.2898 (2)	0.0452 (5)	
C15	0.9459 (2)	0.3837 (3)	−0.1741 (2)	0.0540 (6)	
H15	0.9985	0.3223	−0.1485	0.065*	
C16	0.8865 (2)	0.4463 (3)	−0.0981 (2)	0.0504 (6)	
H16	0.8984	0.4270	−0.0202	0.060*	

### cis-Bicyclo[5.1.0]octan-4-yl 4-bromobenzenesulfonate (II) Atomic displacement parameters (Å^2^)

**Table:**

	*U*^11^	*U*^22^	*U*^33^	*U*^12^	*U*^13^	*U*^23^
Br	0.0708 (3)	0.0710 (3)	0.0602 (2)	0.00970 (14)	0.02049 (16)	−0.01377 (14)
S	0.0477 (3)	0.0424 (3)	0.0456 (3)	−0.0021 (2)	0.0114 (3)	−0.0016 (2)
O1	0.0852 (14)	0.0547 (11)	0.0426 (9)	−0.0235 (10)	0.0260 (9)	−0.0096 (8)
O2	0.0678 (12)	0.0560 (11)	0.0553 (11)	−0.0186 (9)	0.0149 (9)	−0.0152 (9)
O3	0.0680 (13)	0.0787 (14)	0.0725 (13)	0.0296 (11)	0.0124 (11)	0.0048 (11)
C1	0.087 (2)	0.0559 (16)	0.0618 (17)	−0.0115 (16)	0.0251 (16)	0.0041 (14)
C2	0.0514 (14)	0.0613 (16)	0.0462 (13)	−0.0001 (12)	0.0133 (11)	−0.0045 (11)
C3	0.0543 (15)	0.0546 (15)	0.0423 (12)	−0.0010 (11)	0.0065 (11)	−0.0022 (11)
C4	0.0563 (14)	0.0484 (13)	0.0359 (11)	−0.0064 (11)	0.0133 (10)	−0.0054 (10)
C5	0.0561 (16)	0.079 (2)	0.0596 (16)	0.0130 (15)	0.0111 (13)	−0.0017 (15)
C6	0.0702 (19)	0.090 (2)	0.0566 (17)	0.0255 (18)	−0.0044 (14)	0.0041 (16)
C7	0.110 (3)	0.064 (2)	0.0631 (19)	0.0213 (18)	0.0215 (19)	0.0184 (14)
C8	0.133 (3)	0.091 (3)	0.0566 (18)	0.016 (2)	0.032 (2)	0.0242 (18)
C11	0.0412 (12)	0.0409 (12)	0.0380 (11)	−0.0032 (9)	0.0055 (9)	0.0019 (9)
C12	0.0560 (14)	0.0528 (14)	0.0424 (12)	0.0110 (12)	0.0018 (11)	0.0061 (11)
C13	0.0668 (17)	0.0610 (16)	0.0354 (12)	0.0088 (13)	0.0048 (12)	0.0040 (11)
C14	0.0456 (13)	0.0468 (13)	0.0440 (12)	−0.0005 (10)	0.0074 (10)	−0.0032 (10)
C15	0.0520 (14)	0.0601 (16)	0.0493 (14)	0.0162 (12)	0.0022 (11)	0.0036 (12)
C16	0.0553 (15)	0.0605 (15)	0.0352 (11)	0.0100 (12)	0.0040 (10)	0.0078 (11)

### cis-Bicyclo[5.1.0]octan-4-yl 4-bromobenzenesulfonate (II) Geometric parameters (Å, º)

**Table:**

Br—C14	1.892 (2)	C5—C6	1.542 (4)
Br—O2^i^	3.2301 (19)	C5—H5A	0.9700
S—O3	1.421 (2)	C5—H5B	0.9700
S—O2	1.426 (2)	C6—C7	1.502 (5)
S—O1	1.5486 (18)	C6—H6A	0.9700
S—C11	1.755 (2)	C6—H6B	0.9700
O1—C4	1.502 (3)	C7—C8	1.489 (5)
O2—Br^ii^	3.2301 (19)	C7—H7	0.9800
C1—C2	1.485 (4)	C8—H8A	0.9700
C1—C8	1.499 (5)	C8—H8B	0.9700
C1—C7	1.493 (5)	C11—C12	1.379 (3)
C1—H1	0.9800	C11—C16	1.382 (3)
C2—C3	1.534 (3)	C12—C13	1.376 (4)
C2—H2A	0.9700	C12—H12	0.9300
C2—H2B	0.9700	C13—C14	1.368 (4)
C3—C4	1.510 (4)	C13—H13	0.9300
C3—H3A	0.9700	C14—C15	1.387 (4)
C3—H3B	0.9700	C15—C16	1.374 (4)
C4—C5	1.500 (4)	C15—H15	0.9300
C4—H4	0.9800	C16—H16	0.9300
			
C14—Br—O2^i^	170.06 (8)	H5A—C5—H5B	107.9
O3—S—O2	117.52 (14)	C7—C6—C5	113.3 (3)
O3—S—O1	110.01 (14)	C7—C6—H6A	108.9
O2—S—O1	109.64 (11)	C5—C6—H6A	108.9
O3—S—C11	108.87 (12)	C7—C6—H6B	108.9
O2—S—C11	109.64 (11)	C5—C6—H6B	108.9
O1—S—C11	99.66 (10)	H6A—C6—H6B	107.7
C4—O1—S	120.40 (15)	C8—C7—C1	60.3 (2)
S—O2—Br^ii^	107.81 (9)	C8—C7—C6	120.9 (3)
C2—C1—C8	121.6 (3)	C1—C7—C6	119.6 (3)
C2—C1—C7	119.5 (3)	C8—C7—H7	115.0
C8—C1—C7	59.7 (2)	C1—C7—H7	115.0
C2—C1—H1	115.0	C6—C7—H7	115.0
C8—C1—H1	115.0	C7—C8—C1	60.0 (2)
C7—C1—H1	115.0	C7—C8—H8A	117.8
C1—C2—C3	112.9 (2)	C1—C8—H8A	117.8
C1—C2—H2A	109.0	C7—C8—H8B	117.8
C3—C2—H2A	109.0	C1—C8—H8B	117.8
C1—C2—H2B	109.0	H8A—C8—H8B	114.9
C3—C2—H2B	109.0	C12—C11—C16	120.7 (2)
H2A—C2—H2B	107.8	C12—C11—S	120.07 (19)
C4—C3—C2	113.1 (2)	C16—C11—S	119.14 (17)
C4—C3—H3A	109.0	C13—C12—C11	119.3 (2)
C2—C3—H3A	109.0	C13—C12—H12	120.3
C4—C3—H3B	109.0	C11—C12—H12	120.3
C2—C3—H3B	109.0	C12—C13—C14	119.9 (2)
H3A—C3—H3B	107.8	C12—C13—H13	120.0
O1—C4—C5	105.9 (2)	C14—C13—H13	120.0
O1—C4—C3	105.10 (19)	C15—C14—C13	121.2 (2)
C5—C4—C3	118.5 (2)	C15—C14—Br	118.45 (19)
O1—C4—H4	109.0	C13—C14—Br	120.35 (18)
C5—C4—H4	109.0	C14—C15—C16	118.9 (2)
C3—C4—H4	109.0	C14—C15—H15	120.6
C4—C5—C6	112.2 (2)	C16—C15—H15	120.6
C4—C5—H5A	109.2	C11—C16—C15	120.0 (2)
C6—C5—H5A	109.2	C11—C16—H16	120.0
C4—C5—H5B	109.2	C15—C16—H16	120.0
C6—C5—H5B	109.2		
			
C1—C2—C3—C4	−81.7 (3)	C5—C6—C7—C1	−67.2 (4)
C7—C6—C5—C4	80.7 (4)	C2—C1—C7—C6	0.6 (5)
C2—C3—C4—C5	64.4 (3)	C2—C1—C7—C8	111.6 (3)
C6—C5—C4—C3	−63.4 (4)	C6—C7—C1—C8	−110.9 (4)
C3—C2—C1—C7	66.1 (4)		

Symmetry codes: (i) −*x*+2, *y*−1/2, −*z*−1/2; (ii) −*x*+2, *y*+1/2, −*z*−1/2.
